# Variability in statin-induced changes in gene expression profiles of pancreatic cancer

**DOI:** 10.1038/srep44219

**Published:** 2017-03-09

**Authors:** Helena Gbelcová, Silvie Rimpelová, Tomáš Ruml, Marie Fenclová, Vítek Kosek, Jana Hajšlová, Hynek Strnad, Michal Kolář, Libor Vítek

**Affiliations:** 1 Institute of Medical Biology, Genetics and Clinical Genetics, Faculty of Medicine, Comenius University, Bratislava, Slovakia; 2 Department of Biochemistry and Microbiology, University of Chemistry and Technology, Prague, Czech Republic; 3 Laboratory of Genomics and Bioinformatics, Institute of Molecular Genetics, Academy of Sciences of the Czech Republic, Prague, Czech Republic; 4 Institute of Medical Biochemistry and Laboratory Diagnostics, and 4th Department of Internal Medicine, 1st Faculty of Medicine, Charles University, Prague, Czech Republic

## Abstract

Statins, besides being powerful cholesterol-lowering drugs, also exert potent anti-proliferative activities. However, their anti-cancer efficacy differs among the individual statins. Thus, the aim of this study was to identify the biological pathways affected by individual statins in an *in vitro* model of human pancreatic cancer. The study was performed on a human pancreatic cancer cell line MiaPaCa-2, exposed to all commercially available statins (12 μM, 24 h exposure). DNA microarray analysis was used to determine changes in the gene expression of treated cells. Intracellular concentrations of individual statins were measured by UPLC (ultra performance liquid chromatography)-HRMS (high resolution mass spectrometer). Large differences in the gene transcription profiles of pancreatic cancer cells exposed to various statins were observed; cerivastatin, pitavastatin, and simvastatin being the most efficient modulators of expression of genes involved namely in the mevalonate pathway, cell cycle regulation, DNA replication, apoptosis and cytoskeleton signaling. Marked differences in the intracellular concentrations of individual statins in pancreatic cancer cells were found (>11 times lower concentration of rosuvastatin compared to lovastatin), which may contribute to inter-individual variability in their anti-cancer effects. In conclusion, individual statins exert different gene expression modulating effects in treated pancreatic cancer cells. These effects may be partially caused by large differences in their bioavailability. We report large differences in gene transcription profiles of pancreatic cancer cells exposed to various statins. These data correlate to some extent with the intracellular concentrations of statins, and may explain the inter-individual variability in the anti-cancer effects of statins.

Statins, inhibitors of 3-hydroxy-3-methylglutaryl-coenzyme A (HMG-CoA) reductase (Fig. 1), represent the dominant class of compounds for treatment of hypercholesterolemia due to their ability to inhibit *de novo* cholesterol synthesis. In addition to their hypolipidemic effects, owing to depletion of the mevalonate pathway products, statins also exert many other pleiotropic biological activities, preventing the progression of diseases associated with inflammation, increased oxidative stress, and proliferation^1^. Since the introduction of lovastatin as the first novel cholesterol-lowering drug in 1980’s, our understanding of the biological activities of statins has dramatically changed. The potential anti-cancer effects of statins were experimentally demonstrated as early as 1985^2^. Since then, a number of experimental as well as clinical studies, demonstrating the apparent effect of statins on cell proliferation of a variety of tumors have been published (for comprehensive reviews, see refs 1,3). Although multiple biological pathways contribute to the anti-proliferative effects of statins, inhibition of protein prenylation (a critical event in the posttranslational modulation of proteins involved in the regulation of cell cycle progression, proliferation, and signaling pathways) seems to be the most important^4^. Among many protein targets, activation of the Ras protein *via* farnesylation is a key step in cell proliferation. In fact, activation mutations of the *ras* oncogene are present in about 30% of human cancers, and more than 90% of pancreatic cancers^4^.

The majority of clinical data on the potential anti-cancer effects of statins is based on extensive cardiovascular studies. As far as pancreatic cancer, some of these studies have indeed demonstrated a significantly decreased incidence of cancer among statin users, despite a relatively short observation period and improper patient selection (the studies were primarily focused on prevention of cardiovascular diseases)^5^,^6^; nevertheless, other data are not supportive^7^,^8^,^9^,^10^. There are many possible reasons for these discrepancies, including methodological bias^11^, socio-economical aspects^12^, as well as possible differences in the biological activities of individual statins^13^. In our previous study^13^, we reported substantial differences in the anti-cancer effects of individual commercially available statins, and speculated on the possible reasons for these observations.

The aim of this present study was to assess the gene expression profiles in human pancreatic cancer cells bearing an activation mutation in the *ras* oncogene, which were exposed to individual statins.

## Materials and Methods

### Materials

In all experiments, pure forms (≥98%) of the following statins were used: atorvastatin, lovastatin, simvastatin, fluvastatin, cerivastatin, pravastatin, rosuvastatin, and pitavastatin (Alexis; San Diego, CA, USA). All statins were tested in 12 μM concentrations, representing the IC_50_ value for simvastatin after a 24 h treatment of MiaPaCa-2 cancer cells; simvastatin was chosen as the most effective clinically used statin tested *in vitro* in our previous study^13^. All statins were dissolved in methanol.

### Cell culture

Human pancreatic cancer cell line MiaPaCa-2 (ATCC, Manassas, VA, USA), bearing an activation mutation in the *ras* oncogene was maintained in the exponential phase of growth in DMEM medium supplemented with 10% fetal bovine serum in a humidified atmosphere containing 5% CO_2_ at 37 °C. The final concentration of methanol, which was used for dissolving statins, was below 1%. The cell line was authenticated at ATCC by STR profiling before distribution, and also reauthenticated by the end of study by external laboratory (Generi Biotech, Hradec Kralove, Czech Republic).

### Cell growth and viability assessment

The *in vitro* effects of individual statins (pravastatin, atorvastatin, simvastatin, lovastatin, cerivastatin, rosuvastatin, and fluvastatin) on the viability of human pancreatic cancer cells were evaluated in Gbelcová *et al*.^13^. Here, we have assessed the potential anti-proliferative effect of pitavastatin by the same procedure using MiaPaCa-2 cells. The quantity of 2.7 · 10^5^ cells per mL was used for inoculation of individual wells in 6-well plates (total media volume of 2 mL). After 24 h of incubation, the cells were treated with pitavastatin (10, 20, 30 and 40 μM concentrations) dissolved in fresh cell culture media; untreated cells and cells only treated with the vehicle (methanol) served as controls. After 24 h, the medium was removed, the cells were gently washed with PBS, detached from the plate surface by 0.25% trypsin-EDTA solution, and resuspended. Cell growth and viability were determined by direct counting under an inverse microscope using the 0.4% trypan blue exclusion test.

### Determination of intracellular concentrations of statins

MiaPaCa-2 pancreatic cancer cells were exposed to individual statins (12 μM) for 24 h. The cells were then scraped and homogenized in isopropanol to precipitate proteins. After centrifugation, an aliquot of supernatant was used for target analyte quantification (UPLC, Dionex UltiMate^®^ 3000 RSLC; Thermo Scientific, CA, USA), coupled to a HRMS with a Q-orbitrap mass analyzer (Q-ExactiveTM; Thermo Scientific) with a heated electrospray ion source. An Acquity BEH C18 (1.7 μm, 2.1 mm × 100 mm; Waters, MA, USA) separation column was used for chromatographic separation of sample components (mobile phase A consisting of 5 mM ammonium formate and 0.1% formic acid in water:methanol (95:5, v/v); mobile phase B consisted of 5 mM aqueous ammonium formate and 0.1% in 2-propanol:methanol:water (65:30:5, v/v)). To assess the impact of the intracellular concentration of statins on the cancer cell proliferation, a sample of the cancer cells treated with respective statin was used for determination of the intracellular concentration of statins. A parallel cancer cell sample cultured and treated under identical conditions was used to assess the cancer cell proliferation (measured by WST-1 test, Sigma-Aldrich, St Louis, MO, USA).

The quantitation was done by external standard calibration curve (as standards, all commercially available statins were used as described above). Lower limits of quantitation (LLOQ) ranged from 1 to 20 ng/mL, the linear dynamic range was 1000 ng/mL for all analytes. Relative standard deviations did not exceed 11%. As a quality control, the following procedure was used. Statin standards of a known concentration (100 ng/mL) were added to a defined number of otherwise untreated pancreatic cancer cells, which were scraped after 30 min incubation, processed in an identical way as described above, and individual statins were measured in each control cells.

### DNA Microarray Analysis

MiaPaCa-2 pancreatic cancer cells (1.5 × 10^5^ per mL) were used for inoculation of three parallel cultures (10 cm^2^ culture dish, total media volume equal to 10 mL). After 24 h of incubation the cells were treated with statins (12 μM concentration), and dissolved in fresh cell culture media; untreated cells, and cells treated only with the solvent (methanol) served as controls. The cells were further incubated for 24 h. Then, the cells were lysed in the stage of subconfluency using the RLT lysis buffer supplied in the RNeasy Mini Kit (Qiagen, CA, USA). Total RNA was isolated by RNeasy Micro Kit (Qiagen) according to the procedure for animal cells. The quantity of the RNA was measured by a NanoDrop ND-1000 spectrophotometer (NanoDrop Technologies LLC, DE, USA). The quality of the RNA was analyzed using an Agilent 2100 Bioanalyser (Agilent Technologies, CA, USA). RNA samples that had a RIN (RNA integrity number) above 9 were used for further analysis.

Illumina HumanWG-6_V3 Expression BeadChips (Illumina, CA, USA) were used for the microarray analysis, following the standard protocol. In brief, 150 ng of RNA was amplified with an Illumina TotalPrep RNA Amplification Kit (Ambion, TX, USA), and 1.5 μg of labeled RNA was hybridized on the chip according to the manufacturer’s procedure. The analysis was performed in at least two replicates per group (see Suppl. Table 1). To control physiological consistency of the results, the additional Petri-dish replicates for two groups (control and simvastatin) were used, and parallel experiments with 6 μM concentration for all statin groups were also performed. In addition, the technical quality of the microarray data was controlled by technical replicates (see Suppl. Table 1).

The raw data were preprocessed using GenomeStudio software (version 1.9.0.24624; Illumina, CA, USA), and analyzed within the limma package of the Bioconductor as described elsewhere^14^. In short, the transcription profiles were background corrected using a normal-exponential model, quantile normalized and variance stabilized using a base 2 logarithmic transformation.

A moderated t-test was used to detect transcripts differentially expressed between the treated samples and the controls. Those transcripts with a false discovery rate smaller than 0.05, and a fold change smaller than 0.5 or higher than 2, were reported and used in the downstream analysis. The MIAME (Minimum Information About a Microarray Experiment) compliant transcription data was deposited in the ArrayExpress database (accession E-MTAB-3979). Further, we performed the gene set enrichment analysis (GSEA) on KEGG (Kyoto encyclopedia of genes and genomes) pathways^15^ using the Fisher’s exact test and the approach published by Tian *et al*.^16^.

### Quantitative real-time PCR

Reverse transcription was performed using a QuantiTect Reverse Transcription Kit (Qiagen). All experiments were performed in two replicates with all statins (except for rosuvastatin and pravastatin, the least efficient statins from the microarray analyses). The RT-PCR was performed on LightCycler 2.0 System using a LightCycler 480 DNA SYBR Green I Master kit (Roche Diagnostics, Germany) and analyzed by LightCycler software. The resulting crossing point values were normalized using reference genes RPS9, TBP, and GAPDH. Relative fold changes of expression intensity in statin-treated against control samples were computed under the assumption of perfect effectivity of the PCR amplification. Statistical significance was estimated using Student’s t-test. All computations were performed within the R environment^17^. The list of amplicons/primers of randomly selected target and housekeeping genes are provided in Suppl. Table 2.

### STITCH analysis

A functional association network predicted for all eight commercially available statins was created using an interaction network database for small molecules and proteins (based on STRING), STITCH 4.0 (Search Tool for Interaction of Chemicals)^18^. Individual input nodes were atorvastatin, cerivastatin, fluvastatin, lovastatin, pitavastatin, pravastatin, rosuvastatin, and simvastatin. The action view diagrams were generated to illustrate the known protein-chemical relationships of all connected nodes. The view of the statin association network was generated for *Homo sapiens* according to known and predicted interactions including direct (physical) and indirect (functional) associations derived from genomic contexts, high-throughput experiments, co-expression, and literature mining. The confidence score was set to high, equal to 0.850, with a maximum of 150 interactions. The line thickness indicates the confidence score; individual colors indicate the type of the interaction: binding in blue (a ball), activation in green (arrow), inhibition in red (a perpendicular stripe), catalysis in magenta (a ball), the same activity in cyan, and reaction in black (a ball). Individual nodes were clustered based on the Markov clustering algorithm (MCL, inflation equal to 4)^19^.

## Results

### Relationships among cell viability, statin penetrance, and gene expressions in MiaPaCa-2 pancreatic cancer cells exposed to individual statins

**Figure 2:**
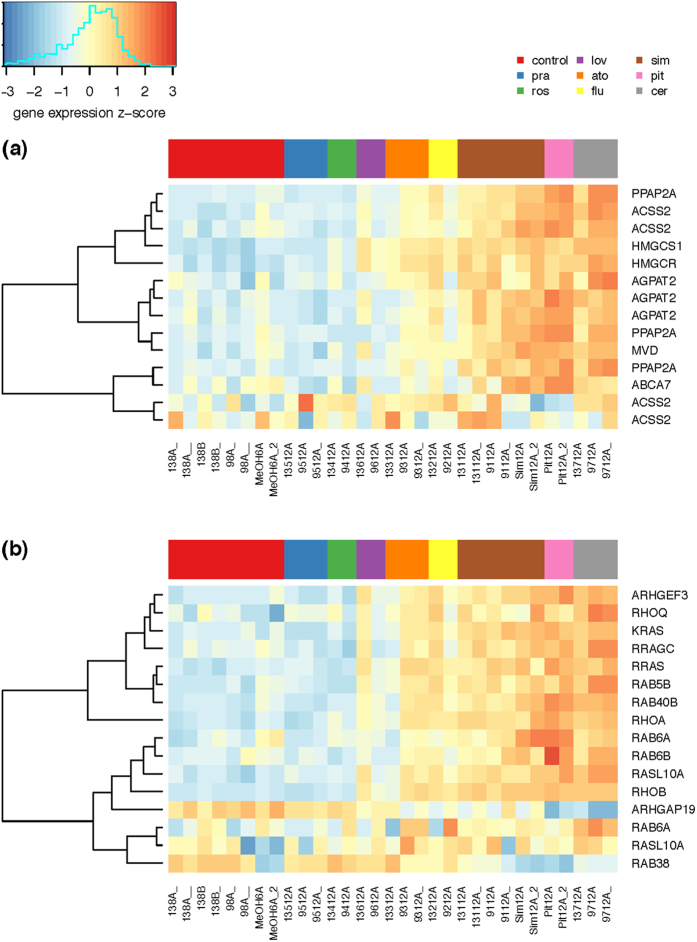
The effect of statins on expression of genes (**a**) related to lipid metabolism, and (**b**) encoding Ras and Ras-related proteins in MiaPaCa-2 pancreatic cancer cells. Figure represents heatmaps of z-score of the log expression intensities of differentially expressed genes. Presented are only the genes with statistically significant difference (FDR < 0.05) in expression intensity in at least one comparison statin vs. control and at least two-fold change of the expression intensity after the statin exposure.

**Figure 3:**
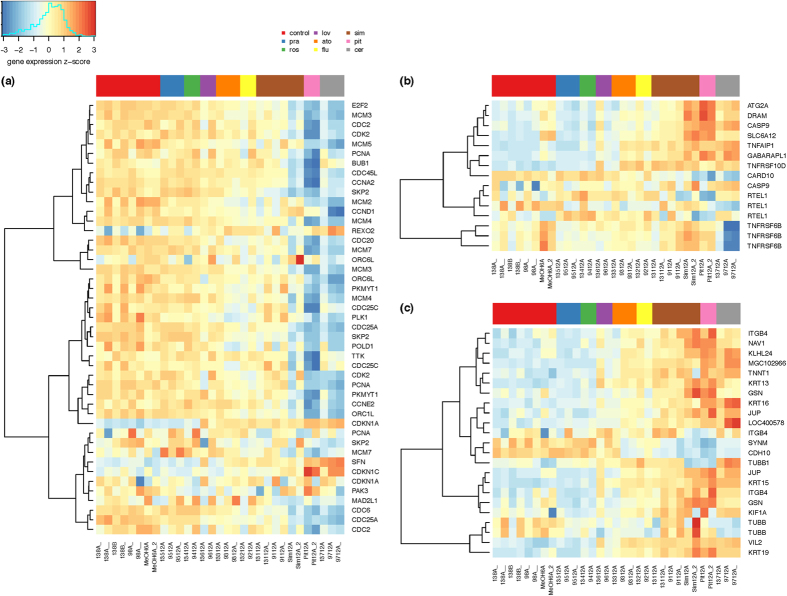
The effect of statins on expression of genes involved in (**a**) cell cycle and DNA replication, (**b**) cell death, and (**c**) cytoskeleton signaling in MiaPaCa-2 pancreatic cancer cells. Figure represents heatmaps of z-score of the log expression intensities of differentially expressed genes. Presented are only the genes with statistically significant difference (FDR < 0.05) in expression intensity in at least one comparison statin vs. control and at least two-fold change of the expression intensity after the statin exposure.

**Figure 4:**
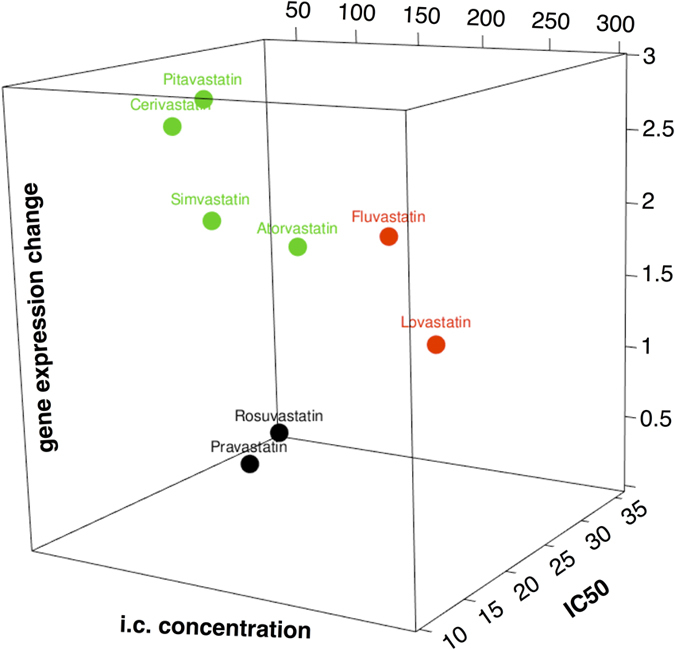
The relationships among the lipophilicity, intracellular concentrations of individual statins, and their impact the gene expression change. Statins were clustered according to their lipophilicity^51^ into 3 groups: hydrophilic (black color), lipophilic (red color), and highly lipophilic (green color). IC50 is expressed in μM, intracellular concentration (i.c.) of statins in nmol/100 000 cells, and gene expression change as log10(#DEG + 1).

Since IC_50_ values for individual statins differ substantially^13^, and this is also true for pitavastatin (Table 1), we were interested in how these differences are reflected in the changes in mRNA expressions. Overall numbers of genes with significant changes in expression (±2 log fold change), after treatment of MiaPaCa-2 cells with individual statins at 12 μM concentrations are listed in Table 1 (for a complete list of the differentially expressed genes see Suppl. Table 3A,B, and also Figs 2 and 3). Consistency of the microarray results can be viewed in the ArrayExpress database, accession E-MTAB-3979 in the heatmaps of all differentially expressed genes in all employed samples (Suppl. Figs 1 and 2). The most effective statins were cerivastatin and pitavastatin; whereas pravastatin and rosuvastatin exhibited practically no effect. The effectiveness of statins on the change in gene expression was lowest in the least lipophilic statins; this observation also correlated well with the anti-proliferative effectiveness of statins on pancreatic cancer cells *in vitro* (Table 1, Fig. 4)^13^. The only exception was lovastatin, which did not have any major effect on gene expression despite its potent anti-proliferative activity *in vitro*.

To assess whether the biological effects of statins correlate with their bioavailability in the treated cells, we determined the intracellular concentrations of statins after their exposure to MiaPaCa-2 pancreatic cancer cells (Table 1). The bioavailability of statins differed substantially, reaching 301.1 nmol/100 000 cells for lovastatin, but only 26.6 nmol/100 000 cells for rosuvastatin (the least efficient anti-proliferative statin) (Table 1, Fig. 4). Interestingly, we were able to find a certain correlation between intracellular levels of statins and their anti-proliferative effects. This correlation was strongest for the least efficient statins (rosuvastatin, pitavastatin and pravastatin); whereas for the most bioavailable statins (in particular lovastatin) this correlation was not so strong (Table 1, Fig. 4). The effects of statins on overall gene expression correlated with their effects on viability and bioavailability only to limited extent. This was especially true for cerivastatin, but not for pitavastatin or lovastatin (Table 1, Fig. 4), suggesting that other factors must play a role in the inter-individual differences in the effects of statins on proliferation of the pancreatic cancer cells used.

### Variability in gene expressions in MiaPaCa-2 pancreatic cancer cells exposed to individual statins

As mentioned above, except for pravastatin and rosuvastatin (the least effective statins *in vitro*), all tested compounds affected the expression of a significant number of genes involved in multiple cell pathways (Table 2, for a complete list of the differentially expressed genes see Suppl. Table 3A,B). As expected, statins significantly modulated the expressions of genes in the mevalonate pathway in treated pancreatic cancer cells (Fig. 2a). The most upregulated gene in this pathway was *HMGCS1* (3-hydroxy-3-methylglutaryl-CoA synthase 1) (Fig. 2a), which encodes the enzyme catalyzing condensation of acetyl-CoA, with acetoacetyl-CoA forming HMG-CoA. Another upregulated gene, *HMGCR* (3-hydroxy-3-methylglutaryl-coenzyme A reductase) is responsible for conversion of HMG-CoA into mevalonic acid. MVD (mevalonate decarboxylase), encoded by the third most affected gene of this pathway, catalyzes the conversion of mevalonate pyrophosphate into isopentenyl pyrophosphate. The intensity of effect of statins on the expression of the above-mentioned genes, related to the mevalonate pathway, decreased in the following order: cerivastatin, pitavastatin, simvastatin, fluvastatin, atorvastatin, lovastatin, pravastatin, and rosuvastatin.

Moreover, cerivastatin, pitavastatin and simvastatin (the most effective anti-proliferative statins) also affected the expression of genes involved in the Kennedy pathway (Fig. 2a) of phospholipid and glycerolipid *de novo* synthesis. These are involved in many biological processes including proliferation, inflammation, carcinogenesis, apoptosis, necrosis, growth arrest, and lipid droplets metabolism^20^. It is interesting to note that pitavastatin compared to most of the other statins affected a number of mevalonate pathway genes despite its low intracellular concentrations (Tables 1 and 2).

Isoprenoids produced in the mevalonate pathway are required for posttranslational modifications of many proteins. As we reported previously, statins affect K-Ras protein trafficking in the pancreatic cancer cells^13^. Therefore, we expected a modulating effect of the statins on the expression of genes encoding the Ras and Ras-related proteins. Indeed, we were able to demonstrate that cerivastatin, pitavastatin, simvastatin, and fluvastatin significantly increased the expressions of *Kras* and also *Rras* genes (except for fluvastatin; Fig. 2b), which promote the formation of focal adhesions, cell spreading, and integrin activation^21^. Moreover, upregulation of several genes belonging to this family of growth regulators was also observed (Fig. 2b). *RhoB*, which was the most dramatically upregulated gene by all statins with the exception of rosuvastatin and pitavastatin (Fig. 2b), belongs to the Rho protein family involved in regulating diverse cellular processes including cytoskeletal organization, gene transcription, cell cycle progression, and cytokinesis^22^. Although the RhoA, upregulated by the three most effective statins, shares 86% amino acid sequence identity with RhoB, RhoB displays several distinct properties such as subcellular localization in endosomes and the pre-lysosomal compartment^23^, rapid turnover of mRNA and protein level^24^, posttranslational modification by either farnesylation or geranylgeranylation^25^, and early upregulation by stress or growth factors^26^. Lastly, while most Rho proteins play a significant role in the stimulation of cell proliferation and malignant transformation processes, RhoB appears instead to act as a negative regulator^27^.

In addition, all statins, except for pravastatin and rosuvastatin, upregulated *CDKN1A* (cyclin-dependent kinase inhibitor 1A), encoding the p21 protein (Fig. 3a) related to cell cycle regulation, which binds to and inhibits the activity of cyclin-cyclin dependent kinases (CDK)2, -CDK1, and -CDK4/6 complexes; thus functioning as a regulator of cell cycle progression in the G1 and S phase. But surprisingly, only two statins (cerivastatin and pitavastatin), caused significant downregulation of additional genes encoding proteins involved in cell cycle regulation and DNA replication, among others: *CDC2*, *CDC25A*, *SKP2*, *E2F2*, *CDC3*, or origin recognition complex (*ORC)1L*/*6L* (Fig. 3a); suggesting that cerivastatin and pitavastatin may block cell cycle progression at the S phase, whereas the remaining statins did not exert any significant effect on the expression of these genes.

The other group of genes affected by statin treatment were those involved in apoptosis (Fig. 3b). Similar differential effects on gene expression were also observed for this group of genes, with the *TNFRSF10D* gene being the most differentially expressed; its expression was significantly upregulated independently on the type of statin used (Fig. 3b). The biological relevance of this observation is uncertain, since TNFRSF10D does not induce apoptosis. On the other hand, the upregulation of the *GABARAPL* gene (also known as an early estrogen-regulated protein) (Fig. 3b) suggests that cerivastatin, pitavastatin, simvastatin, and fluvastatin might induce cell death by a different mechanism such as autophagy^28^. Furthermore, both cerivastatin and pitavastatin also increased the expression of the *DRAM* gene (Fig. 3b) implemented in the p53 tumor suppressor and autophagy pathways. Decreased transcriptional expression of this gene is associated with the development of various tumors^29^.

The expressions of genes encoding several cytoskeletal proteins involved in cell-to-cell adhesion were also changed by statin treatment (again, except for pravastatin and rosuvastatin, Fig. 3c). All effective statins upregulated genes encoding keratins or cytokeratins; other genes differentially expressed upon exposure to individual statins involved those coding for junction plakoglobin (*JUP*, gamma-catenin), cadherin 10 (*CDH10*), synemin (desmuslin), kinesins, or ezrin (Fig. 3c).

### Quantitative PCR analysis of selected genes

Although the comparisons of RT-PCR and microarray results were limited by a low number of replicates in the RT-PCR analyses, quantitative PCR analysis of randomly selected genes revealed trends in the gene expressions in agreement with the microarray data obtained (Suppl. Table 4).

### Predicted interactions of statins with their known biological targets

**Figure 5:**
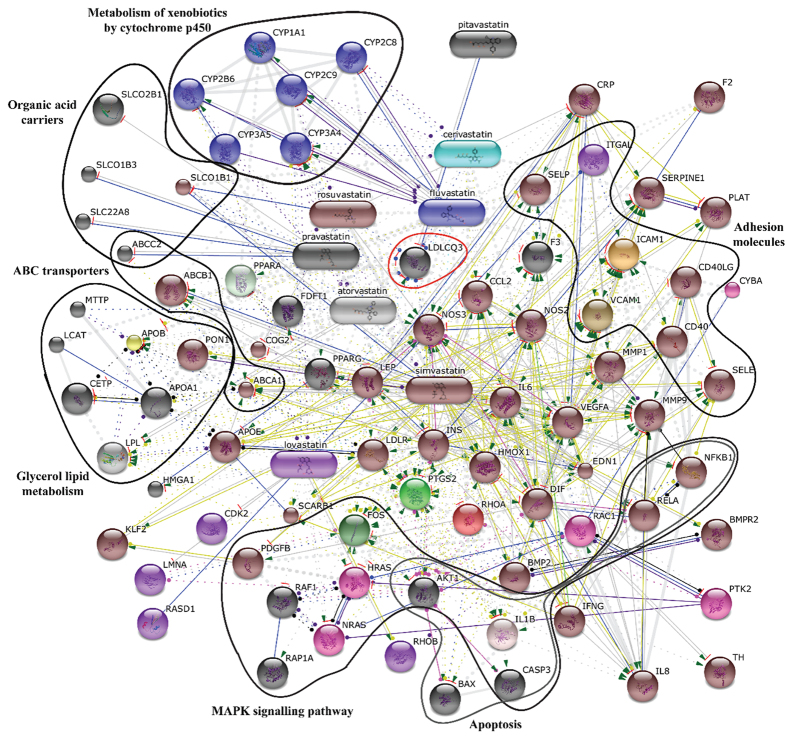
Predicted functional association networks for all statins. Individual nodes represent drugs and genes products. Input nodes: atorvastatin, cerivastatin, fluvastatin, lovastatin, pitavastatin, pravastatin, rosuvastatin, and simvastatin. The action view of the statin association network was generated according to known and predicted interactions for *Homo sapiens*. Individual node colors indicate the type of the interaction: binding – blue ball, activation – green arrow, inhibition – red bar, catalysis – magenta ball, same activity - cyan, reaction – black ball. The line thickness indicates the confidence score. The complete description of the listed genes is stated in Suppl. Table 5.

Finally, we performed an *in silico* analysis of the predicted interactions of all eight commercially available statins with their known biological targets, using the STICH database (Fig. 5). The results were clustered according to the Markov clustering algorithm into four groups. Interestingly, no interaction has previously been reported for pitavastatin for the set confidence score of 0.850. The detected results were compared with the data gained from the microarray analysis. Thus, based on the results from the microarray analysis (selected genes with changed expression in Figs 2 and 3) we extended the information available on a number of genes, expressions of which were affected by pitavastatin treatment (see Figs 2 and 3). The only reported/predicted (inhibition) interaction for pitavastatin so far, has been HMGCR.

## Discussion

Although not all of the published data are conclusive^7^,^8^,^9^,^10^, statins have been demonstrated in several studies to significantly decrease risk of pancreatic cancer^5^,^6^,^30^. In fact, both reduction of overall cancer as well as pancreatic cancer-related mortality rates were reported in a recent large Danish study^31^.

One of the possible reasons, which may account for the reported discrepancies in the outcomes of statin-treated cancer patients might lie in the inter-individual differences among these compounds^13^. Indeed, a recent report showed significant differences among individual statins and their effects on the risk of lung, breast, and hematological cancers^32^. Similarly, use of simvastatin was demonstrated to be more potent, compared to the other statins in terms of the survival of pancreatic cancer patients^33^; simvastatin but not lovastatin improved the survival rate in patients undergoing resection for early-stage pancreatic cancer^34^. On the other hand, lovastatin was the only efficient statin in patients with colorectal cancer^35^. In general, lipophilic statins were superior in their protective functions over hydrophilic compounds in patients with pancreatic^30^ and breast^36^ cancers. All of these data indicate that statins, despite their common inhibitory effect on HMG-CoA (their major intracellular target) exert substantial differences in their biological outcomes.

The data from our comprehensive analyses confirmed large inter-individual variability among all of the clinically-used statins, in terms of their effects on the metabolism and signaling of pancreatic cancer cells. Indeed, we found large differences in gene transcription profiles of pancreatic cancer cells exposed to statins with cerivastatin, pitavastatin, and simvastatin, which were the most efficient modulators of gene expressions. Although to some extent these results correlated with the intracellular concentrations of the statins, to our surprise, this correlation was not as strong as expected. Apart from cerivastatin (the most efficient anti-proliferative statin, and also having the deepest impact on the expression of a wide array of intracellular targets in pancreatic cancer cells) the second most influential statin was pitavastatin, despite its relatively low bioavailability and only moderate anti-proliferative efficiency. Interestingly, pravastatin and rosuvastatin, capable of reaching the intracellular compartment in only very low concentrations, were practically without any significant effect on gene expressions.

It should be noted that the observed results are very likely to be cell type-specific, since completely different results in terms of anti-proliferative activities as well as intracellular concentrations of statins, were reached for both human hepatoblastoma (HepG2) cells and human embryonic kidney cells (our own unpublished data). This is also supported by recent data by Menter *et al*. who demonstrated the differential effects of pravastatin and simvastatin on a number of malignant cell lines^37^.

Apart from the expected impacts of statin treatment on the genes involved in cholesterol metabolism, our results also indicate that some statins (namely cerivastatin and pitavastatin, and to lesser extent also other statins, except for rosuvastatin and pravastatin) directly affect the expression of specific genes related to the Rho GTPase signaling, and cytoskeletal regulation in the pancreatic cancer cell line used. These data are in line with the effect of simvastatin on a similar group of genes observed in endothelial cells^38^. In another DNA microarray study, Johnson-Anuna *et al*. tried to identify gene expression patterns in the cerebral cortex of mice treated with oral doses of simvastatin (50 mg·kg^−1^ b. wt.) once a day for 21 days. They found that simvastatin significantly reduced the expression of the proto-oncogenic gene *c-fos* with simultaneously enhanced expression of both the *c-myc* oncogene and antiapoptotic *Bcl-2* gene^39^. We were not able to confirm this data in our study, presumably due to the different experimental model and also the much lower statin dosage. In our experiments, only cerivastatin increased the expression of a gene encoding for caspase 9 (Fig. 3b), an initiator caspase, which has been linked to the mitochondrial death pathway. Nevertheless, as demonstrated by the analysis of the most altered functional pathways (Table 2, Suppl. Table 3A,B), statins affected some processes related to DNA repair, such as base excision repair (pitavastatin) or mismatch repair (cerivastatin, pitavastatin, simvastatin). It is known that failure of these processes could be followed by programmed necrosis^40^. Concurrently, cerivastatin, simvastatin, pitavastatin, and additionally fluvastatin caused upregulation of *GABARAPL1* gene, related to autophagy (Fig. 3b). Out of many end products of this pathway, ubiquinone is required in the process of ATP formation during oxidative phosphorylation. Interestingly, it was reported that ubiquitinated hydrophobic proteins, which are prone to aggregation, are kept on the surface of lipid droplets (formation of lipid droplets is affected by statins^20^) and subjected to autophagy as well as proteasomal degradation^41^.

The cell cycle arrest induced by statins represents another frequently discussed, potential anti-cancer pathway. Many reports describe the effect of individual statins on the expression of the cell cycle-related genes. For example, changes in the expression of a number of genes related to the cell cycle in chronic myelogenous leukemia K562 cells upon exposure to simvastatin were described^42^. The results of flow cytometry showed that the cell cycle was arrested in the G1 phase^42^. In another study, Assmus B *et al*.^43^ performed a microarray analysis using a primary cell line of endothelial progenitor cells treated with low concentrations of atorvastatin. Among other studies, the expression of genes coding for cyclins and PCNA was increased after atorvastatin incubation; whereas that of the cell cycle inhibitory protein p27 was reduced^43^. Similarly, the downregulation of cyclin D1, *PCNA*, *c-myc*, as well as the upregulation of *p21* and *p19* were reported in human breast cancer cells treated with cerivastatin^44^. In contrast to this study, the expression of genes encoding cyclins or PCNA was not affected by atorvastatin treatment in our microarray analysis, underlining the cell specificity dependent biological variability of individual statins. In fact, only the two most effective statins (cerivastatin and pitavastatin) affected the expression of cyclins and *PCNA*; with both statins inducing downregulation of the mentioned genes (Fig. 3a). Consistent with the reported data^44^, the expression of the *p21* gene was increased by all effective statins (Fig. 3a). On the other hand, the genes associated with DNA replication, such as subunits of the *ORC*, and components of the minichromosome maintenance complex were downregulated by cerivastatin and pitavastatin (Fig. 3a); thus suggesting that cerivastatin and pitavastatin primarily blocked the progress of the cell cycle through the S phase. Likewise, in our study, other proteins related to both the S phase (*SKP2*, *E2F2*) and M phase (*CDC*s) were downregulated by cerivastatin and pitavastatin, indicating that except for the G1 phase, the statins mentioned blocked the cell cycle entry into the M phase. This is not surprising, as lovastatin is used in the cell-cycle synchronization protocols^45^ and as a pharmacological tool for controlling the growth of neoplastic cells^46^. Furthermore, lovastatin is commercially available as an inhibitor of the cell cycle in the G1 and G2/M phase (Sigma-Aldrich). Last but not least, cerivastatin, pitavastatin, simvastatin and fluvastatin also increased the expression of the *Kras* gene implicated in multiple signaling pathways; thus accounting for the pleiotropic effects of the statins. It is likely that upregulation of the *Kras* gene after statin treatment reflects the unavailability of properly posttranslationally modified K-Ras protein for cell signaling due to inhibition of the mevalonate pathway. Similarly, this mechanism could also explain the upregulation of other Ras and Ras-related proteins induced by statin treatment. However, the products of the mevalonate pathway are required not only for posttranslational modifications of various proteins; but also for the functional regulation of posttranslational modifications of intermediate filaments, including nuclear lamins as well as cytoplasmic keratins, vimentin, desmin, glial fibrillary acidic protein, or neurofilaments. In general, the pancreatic cancer cells used in this study were firmly attached to the cultivating surface, and their detachment was very inefficient. However, the treatment of the cells with effective statins changed their shape and facilitated their detachment (data not shown). This is consistent with the effect of statins on the expression of genes encoding the cytoskeletal proteins (Fig. 3c).

It should also be emphasized that statins certainly have broad and variable modulatory effects on gene expressions, which differ substantially among various animal models, as well as among various organs even within the same animals^47^; additionally the same phenomenon of gene expression variability dependent on cell-type specificity, was also confirmed in *in vitro* studies^48^,^49^.

Our study has several limitations. First, it is generally known that transcriptomics data do not always translate into phenotype, most likely due to posttranscriptional modification of encoded proteins or their increased degradation rates^50^. Furthermore, only one human pancreatic cancer cell line was used in our studies, and it is likely that the mRNA transcriptomics profiles would substantially differ even within different pancreatic cancer cells.

In conclusion, we found large differences in gene transcription profiles of pancreatic cancer cells exposed to various statins, with cerivastatin, pitavastatin, and simvastatin being the most efficient modulators of gene expressions. To some extent these results correlated with intracellular concentrations of statins; although this was not the case for pitavastatin, which potently changed the expression of a wide array of genes despite its relatively low bioavailability, and only moderate anti-proliferative efficiency. Our data may account for the inter-individual variability in the anti-cancer effects of individual statins; although further, particularly proteomic studies, would be required to fully uncover this phenomenon.

## Additional Information

**How to cite this article:** Gbelcová, H. *et al*. Variability in statin-induced changes in gene expression profiles of pancreatic cancer. *Sci. Rep.* **7**, 44219; doi: 10.1038/srep44219 (2017).

**Publisher's note:** Springer Nature remains neutral with regard to jurisdictional claims in published maps and institutional affiliations.

## Supplementary Material

Supplementary Table 1

Supplementary Table 3

Supplementary Tables 2, 4 and 5

## Figures and Tables

**Figure 1:**
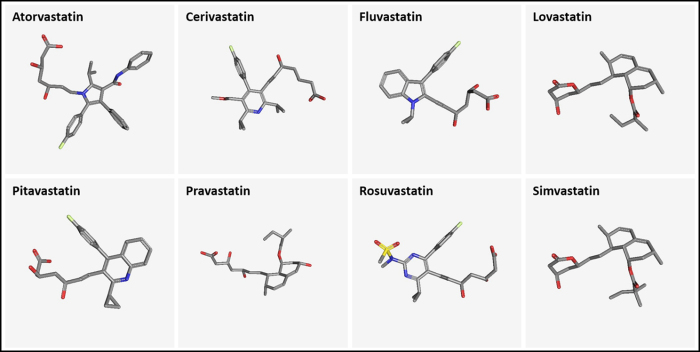
3D conformers of commercially available statins. Grey – carbon, red – oxygen, blue – nitrogen, light green – fluorine, yellow – sulphur.

**Table 1:** The effect of individual statins on viability, cell penetrance, and gene expression in MiaPaCa-2 pancreatic cancer cells.

IC_50_ studies* [24 h exposure, μM]		Gene expression studies	Upregulated genes (No.)	Downregulated genes (No.)	Total genes with changed expression (No.)	i.c. concentration of statin [nmol/100 000 cells]**		Proportion of i.c. statin concentration to that of lovastatin [%]	Lipophilicity of statins***
Cerivastatin	10	Cerivastatin	397	268	665	Lovastatin	301.1	—	Atorvastatin
Simvastatin	12	Pitavastatin	344	320	664	Fluvastatin	189.1	97	Simvastatin
Lovastatin	13	Simvastatin	128	38	166	Simvastatin	156.4	61	Pitavastatin
Pitavastatin	20	Fluvastatin	59	15	74	Cerivastatin	146.3	56	Cerivastatin
Fluvastatin	26	Atorvastatin	41	10	51	Atorvastatin	111.5	52	Fluvastatin
Atorvastatin	27	Lovastatin	33	5	38	Pitavastatin	92.6	36	Lovastatin
Pravastatin	29	Pravastatin	0	0	0	Pravastatin	50.7	25	Pravastatin
Rosuvastatin	36	Rosuvastatin	0	0	0	Rosuvastatin	26.6	18	Rosuvastatin

*Data retrieved from^13^ were combined with those of pitavastatin experiment.

**i.c. statin concentration measured at the end of 24-h incubation and recalculated to 100 000 cells to take into account different antiproliferative potential of individual statins.

***Statins sorted from the most to the least lipophilic compounds. Lipophilicity of ring-opened forms of statins based on partition between n-octanol and water^51^.

Analyses of intracellular (i.c.) concentrations of statins were performed in duplicates after 24 h incubation with respective statin (initial concentration was 12 μM).

Differentially transcribed genes detected in statin-treated cells (12 μM) compared to untreated control samples. Presented are only the transcripts with FC > 2.0 or < 0.5 and FDR < 0.05 (FC – fold change, FDR – false discovery rate). For full list of differentially regulated transcripts see the ArrayExpress database, accession E-MTAB-3979.

**Table 2:** Metabolic and signaling pathways affected by statin treatment of MiaPaCa-2 pancreatic cancer cells.

Path ID	Path name	Cerivastatin	Pitavastatin	Simvastatin	Fluvastatin	Atorvastatin	Lovastatin	Pravastatin	Rosuvastatin
		False discovery rate (FDR)							
hsa00100	Steroid biosynthesis	—	<10^−7^	—	—	—	<10^−9^	—	—
hsa03030	DNA replication	<10^−9^	<10^−9^	<10^−9^	<10^−9^	<10^−8^	<10^−3^	—	—
hsa04110	Cell cycle	<10^−9^	<10^−9^	<10^−8^	<10^−9^	<10^−9^	<10^−6^	—	—
hsa03040	Spliceosome	<10^−9^	<10^−9^	<10^−9^	<10^−9^	<10^−8^	<10^−8^	—	—
hsa03430	Mismatch repair	<10^−9^	<10^−9^	<10^−5^	—	—	—	—	—
hsa03440	Homologous recombination	<10^−8^	<10^−8^	<10^−4^	<10^−4^	—	—	—	—
hsa03020	RNA polymerase	<10^−7^	—	<10^−8^	<10^−5^	<10^−3^	10^−3^	—	—
hsa04144	Endocytosis	<10^−6^	<10^−6^	<10^−8^	<10^−6^	<10^−3^	—	—	—
hsa00240	Pyrimidine metabolism	<10^−6^	<10^−6^	<10^−6^	<10^−4^	<10^−3^	—	—	—
hsa04146	Peroxisome	<10^−5^	<10^−3^	—	<10^−3^	—	—	—	—
hsa03018	RNA degradation	<10^−3^	—	—	—	—	—	—	—
hsa03010	Ribosome	—	—	<10^−8^	10^−3^	—	<10^−3^	—	—
hsa04010	MAPK signaling pathway	—	—	<10^−4^	—	—	<10^−3^	—	—
hsa04540	Gap junction	—	—	<10^−4^	—	—	—	—	—
hsa00230	Purine metabolism	—	—	<10^−4^	—	—	—	—	—
hsa03410	Base excision repair	—	<10^−3^	—	—	—	—	—	—

KEGG pathways enriched for differentially expressed genes (DEG) as detected by GSEA. GSEA was performed individually for each comparison of statin-treated (12 μM) and control samples. FDR < 0.001 was used as a cut-off value.
