# Supplementary material for: Variability in statin-induced changes in gene expression profiles of pancreatic cancer
Source: Sci Rep. 2017 Mar 9;7:44219. doi: 10.1038/srep44219 (PMC5343581; doi:10.1038/srep44219)
Supplement: Supplementary Tables 2, 4 and 5 [file srep44219-s3.doc]

**Gbelcová H, Rimpelová R, Ruml T, Fenclová M, Kosek V, Hajšlová J, Strnad H, Kolář M, Vítek L. Variability in statin-induced changes in gene expression profiles of pancreatic cancer.**

**Supplementary Tables 2, 4 and 5**

**Supplementary Table 2. List of primers us**ed for quantitative real-time PCR analyses

| **Gene** | **Left primer** | **Right primer** | **amplicon nt** |
| --- | --- | --- | --- |
| TUBB1 | TGTACTACAACGAAGCCTACGG | GAGAGCTCCTAATTTGCTAGATCG | 107 |
| RHOB | GCATGAACAGGACTTGACCA | TGTGTCCTCCCCAAGTCAGT | 70 |
| K-RAS | CACTGTTCACAAAGGTTTTGTCTC | TTGGGGAGAGTGACCATGA | 60 |
| HMGCR | CTGGGGAATTGTCACTTATGG | GAGGTCTTGTAAATTGATCTTCGAC | 95 |
| CDH10 | TCAAAACCTCTTGACCGTGA | CGTGTTGTCTCTTTGGGATTG | 86 |
| TBP | GGGGAGCTGTGATGTGAAGT | CCAGGAAATAATTCTGGCTCA | 93 |
| RPS9 | ATCCGCCAACGTCACATTA | TCTTCACTCGGCCTGGAC | 136 |
| GAPDH | GCTCTCTGCTCCTCCTGTTC | ACGACCAAATCCGTTGACTC | 62 |

**Supplementary Table 4. Quantitative PCR analysis of selected genes**

| **Gene** | **Method** | **Cerivastatin** | **Pitavastatin** | **Simvastatin** | **Fluvastatin** | **Atorvastatin** | **Lovastatin** |
| --- | --- | --- | --- | --- | --- | --- | --- |
| ***CDH10*** | RT-qPCR | **0.21** **.** | **0.08 .** | 0.13 n.s. | **0.16 **** | 0.28 n.s. | 0.32 n.s. |
|  | Microarray | **0.31 ***** | **0.27 ***** | **0.33 ***** | **0.48 **** | **0.45 ***** | **0.52 *** |
| ***HMGCR*** | RT-qPCR | **4.5 .** | **5.7 .** | **2.3 .** | 1.3 n.s. | n.a. | n.a. |
|  | Microarray | **3.9 ***** | **3.2 ***** | **3.0 ***** | **2.7 ***** | **2.4 ***** | **2.1 ***** |
| ***Kras*** | RT-qPCR | **3.7 *** | **4.2 *** | 2.6 n.s. | 2.0 n.s. | n.a. | n.a. |
|  | Microarray | **3.2 ***** | **2.6 ***** | **2.6 ***** | **2.0 ***** | **1.9 ***** | **1.7 *** |
| ***RhoB*** | RT-qPCR | **40 *** | **42 *** | **24 ***** | 17 n.s. | 9.5 n.s. | 8.5 n.s. |
|  | Microarray | **13 ***** | **12 ***** | **11 ***** | **7.1 ***** | **5.6 ***** | **4.9 ***** |
| ***TUBB1*** | RT-qPCR | **5.0 .** | n.a. | n.a. | n.a. | n.a. | n.a. |
|  | Microarray | **4.0 ***** | **1.6 *** | **1.7 ***** | **2.2 ***** | **1.7 **** | n.a. |

Fold expression changes in statin treated samples vs. controls as detected in RT-qPCR and microarrary analyses. Figures in bold denote statistically significant changes (p < 0.1 for RT-qPCR, and FDR < 0.1 for microarray data). The symbols denote: n.a. not available, n.s. not significant, **.** p (resp. FDR) < 0.1, ***** p < 0.05, ****** p < 0.01, ******* p < 0.001.

**Supplementary Table 5. List of genes and respective gene products from the STITCH analysis**

| **Gene** | **Gene product** |
| --- | --- |
| ***ABCA1*** | ATP-binding cassette, sub-family A (ABC1), member 1 |
| ***ABCB1*** | ATP-binding cassette, sub-family B (MDR/TAP), member 1 |
| ***ABCC2*** | ATP-binding cassette, sub-family C (CFTR/MRP), member 2 |
| ***AKT1*** | v-akt murine thymoma viral oncogene homolog 1 |
| ***APOA1*** | apolipoprotein A-I |
| ***APOB*** | apolipoprotein B (including Ag(x) antigen) |
| ***APOE*** | apolipoprotein E |
| ***BAX*** | BCL2-associated X protein |
| ***BMP2*** | bone morphogenetic protein 2 |
| ***BMPR2*** | bone morphogenetic protein receptor, type II (serine/threonine kinase) |
| ***CASP3*** | caspase 3, apoptosis-related cysteine peptidase |
| ***CCL2*** | chemokine (C-C motif) ligand 2 |
| ***CD40*** | CD40 molecule, TNF receptor superfamily member 5 |
| ***CD40LG*** | CD40 ligand |
| ***CDK2*** | cyclin-dependent kinase 2 |
| ***+CETP*** | cholesteryl ester transfer protein, plasma |
| ***COG2*** | component of oligomeric golgi complex 2 |
| ***CRP*** | C-reactive protein, pentraxin-related |
| ***CYBA*** | cytochrome b-245, alpha polypeptide |
| ***CYP1A1*** | cytochrome P450, family 1, subfamily A, polypeptide 1 |
| ***CYP2B6*** | cytochrome P450, family 2, subfamily B, polypeptide 6 |
| ***CYP2C8*** | cytochrome P450, family 2, subfamily C, polypeptide 8 |
| ***CYP2C9*** | cytochrome P450, family 2, subfamily C, polypeptide 9 |
| ***CYP3A4*** | cytochrome P450, family 3, subfamily A, polypeptide 4 |
| ***CYP3A5*** | cytochrome P450, family 3, subfamily A, polypeptide 5 |
| ***DIF*** | Tumor necrosis factor Precursor (TNF-alpha) (Tumor necrosis factor ligand superfamily member 2) |
| ***EDN1*** | endothelin 1 |
| ***F2*** | coagulation factor II (thrombin) |
| ***F3*** | coagulation factor III (thromboplastin, tissue factor) |
| ***FDFT1*** | farnesyl-diphosphate farnesyltransferase 1 |
| ***FOS*** | FBJ murine osteosarcoma viral oncogene homolog |
| ***HMGA1*** | high mobility group AT-hook 1 |
| ***HMOX1*** | heme oxygenase (decycling) 1 |
| ***HRAS*** | v-Ha-ras Harvey rat sarcoma viral oncogene homolog |
| ***ICAM1*** | intercellular adhesion molecule 1 |
| ***IFNG*** | interferon, gamma |
| ***IL1B*** | interleukin 1, beta |
| ***IL6*** | interleukin 6 (interferon, beta 2) |
| ***IL8*** | interleukin 8 |
| ***INS*** | insulin |
| ***ITGAL*** | integrin, alpha L (antigen CD11A (p180), lymphocyte function-associated antigen 1 |
| ***KLF2*** | Kruppel-like factor 2 (lung) |
| ***LCAT*** | lecithin-cholesterol acyltransferase |
| ***HMGCR*** | 3-hydroxy-3-methylglutaryl-Coenzyme A reductase |
| ***LDLR*** | low density lipoprotein receptor |
| ***LEP*** | leptin |
| ***LMNA*** | lamin A/C |
| ***LPL*** | lipoprotein lipase |
| ***MMP1*** | matrix metallopeptidase 1 (interstitial collagenase) |
| ***MMP9*** | matrix metallopeptidase 9 (gelatinase B, 92kDa gelatinase, 92kDa type IV collagenase) |
| ***MTTP*** | microsomal triglyceride transfer protein |
| ***NFKB1*** | nuclear factor of kappa light polypeptide gene enhancer in B-cells 1 |
| ***NOS2*** | nitric oxide synthase 2, inducible |
| ***NOS3*** | nitric oxide synthase 3 (endothelial cell) |
| ***NRAS*** | neuroblastoma RAS viral (v-ras) oncogene homolog |
| ***PDGFB*** | platelet-derived growth factor beta polypeptide (simian sarcoma viral (v-sis) oncogene homolog) |
| ***PLAT*** | plasminogen activator, tissue |
| ***PON1*** | paraoxonase 1 |
| ***PPARA*** | peroxisome proliferator-activated receptor alpha |
| ***PPARG*** | peroxisome proliferator-activated receptor gamma |
| ***PTGS2*** | prostaglandin-endoperoxide synthase 2 (prostaglandin G/H synthase and cyclooxygenase) |
| ***PTK2*** | PTK2 protein tyrosine kinase 2 |
| ***RAC1*** | ras-related C3 botulinum toxin substrate 1 (rho family, small GTP binding protein Rac1) |
| ***RAF1*** | v-raf-1 murine leukemia viral oncogene homolog 1 |
| ***RAP1A*** | RAP1A, member of RAS oncogene family |
| ***RASD1*** | RAS, dexamethasone-induced 1 |
| ***RELA*** | v-rel reticuloendotheliosis viral oncogene homolog A (avian) |
| ***RHOA*** | ras homolog gene family, member A |
| ***RHOB*** | *ras* homolog gene family, member B |
| ***SCARB1*** | scavenger receptor class B, member 1 |
| ***SELE*** | selectin E |
| ***SELP*** | selectin P (granule membrane protein 140kDa, antigen CD62) |
| ***SERPINE1*** | serpin peptidase inhibitor, clade E (nexin, plasminogen activator inhibitor type 1), member 1 |
| ***SLCO1B1*** | solute carrier organic anion transporter family, member 1B1 |
| ***SLCO1B3*** | solute carrier organic anion transporter family, member 1B3 |
| ***SLC22A8*** | solute carrier family 22 (organic anion transporter), member 8 |
| ***SLCO2B1*** | solute carrier organic anion transporter family, member 2B1 |
| ***TH*** | tyrosine hydroxylase |
| ***VCAM1*** | vascular cell adhesion molecule 1 |
| ***VEGFA*** | vascular endothelial growth factor A |
